# Novel use of an atrial Aveir AR pacemaker implanted in the right ventricle for a pediatric patient with prolonged sinus pause

**DOI:** 10.1016/j.ipej.2026.03.010

**Published:** 2026-03-09

**Authors:** Kenan S. Wayne, Daniel Cortez

**Affiliations:** Department of Pediatrics, University of California at Davis, Sacramento, USA

## Abstract

Leadless pacemakers offer an attractive alternative to transvenous systems in children but are limited by vascular size, cardiac chamber dimensions, and device-specific characteristics. We describe the first implantation of an atrial Aveir AR leadless pacemaker in the right ventricle of a pediatric patient using a superior venous approach. A 7-year-old, 23.7-kg girl presented with recurrent syncope and was found to have an 8.7-s sinus pause on ambulatory monitoring consistent with sinus node dysfunction. Pre-procedural echocardiography and ultrasound assessment demonstrated relatively small atrial dimensions and identified the right internal jugular vein (1 cm) as the largest available central vessel compared with the femoral and left internal jugular veins. To optimize catheter maneuverability and accommodate the 27-Fr delivery sheath, a right internal jugular approach was selected. The Aveir AR device was advanced across the tricuspid valve and fixed to the mid–right ventricular septum on the first attempt, with stable sensing and capture parameters and no periprocedural complications (threshold 0.5 V at 0.2 ms, impedance 400 Ω, R-wave 5.9 mV). The patient was discharged within 24 hours and remained asymptomatic with preserved device function on one month follow-up, with threshold 0.75 V at 0.2 ms, impedance 410 Ω, R-wave 8.3 mV, and with <1% pacing, predicted longevity at 15.9 years. This case illustrates that superior venous access can facilitate safe ventricular deployment of an Aveir AR leadless pacemaker in select pediatric patients with sinus pauses.

## 1 Introduction

Leadless pacemaker (LPM) technology has evolved significantly since the first device—a Nanostim—was implanted in an adult patient in 2012 [1]. Since that time, two newer systems, the Micra (Medtronic) and the Nanostim successor, Aveir (Abbott), have emerged, and their use has expanded from adults to selected pediatric patients. LPMs offer important advantages over traditional transvenous pacemakers, including the avoidance of lead-related complications such as lead failure, infection, and cosmetic concerns [1]. However, they carry their own procedural risks, including higher rates of pericardial effusion and cardiac perforation [1].

Anatomical differences between pediatric and adult patients are critical when planning LPM implantation. Prior studies have shown that, in children, superior venous access—particularly via the internal jugular veins—may be preferable to femoral access due to relatively larger vessel size, a relationship that reverses with adulthood [2]. Furthermore, the comparatively reduced chamber size in pediatric atria and ventricles can constrain intraprocedural device rotation and hinder the ability to achieve secure fixation. These anatomical considerations underscore the importance of selecting an appropriate device, as LPMs differ in key features such as length, diameter, battery longevity, fixation mechanism, and retrievability [1,[3], [4], [5]].

As LPM technology advances and patient anatomy varies, individualized approaches to implantation are essential; placement is not a “one-size-fits-all” procedure. Here, we report the first known implantation of an Aveir AR LPM in the right ventricle of a pediatric patient weighing 23.7 kg, performed for recurrent syncope secondary to sinus node dysfunction [6].

## 2 Methods

A retrospective review was performed at the University of California at Davis of the atrial chamber leadless pacemaker (Aveir AR) procedure.

## 3 Case

A 7-year-old female was referred to pediatric cardiology for evaluation of recurrent syncopal episodes. Her first episode occurred in January 2022 following a minor hand injury. While being evaluated at a clinic shortly after the injury, she reported feeling dizzy and subsequently lost consciousness. She recovered spontaneously within minutes.

A second episode occurred in March 2024 during routine classroom activities. While seated at her desk, she developed sudden lightheadedness. As she stood and began walking to rotate between group stations, she collapsed. She regained consciousness quickly and returned to baseline without residual symptoms.

A third syncopal event was reported a few months later. The patient was standing by a window at night observing fireflies with her younger sister when she suddenly fainted. The event was unwitnessed by an adult; the patient's mother was asleep at the time, and additional details were unavailable.

The most recent episode occurred at home while her mother was brushing her hair. The patient had been standing for approximately 2 min when she experienced an abrupt loss of consciousness and fell to the floor. Her mother noted that a few minutes earlier the child had complained of mild abdominal discomfort.

**Fig. 1:**
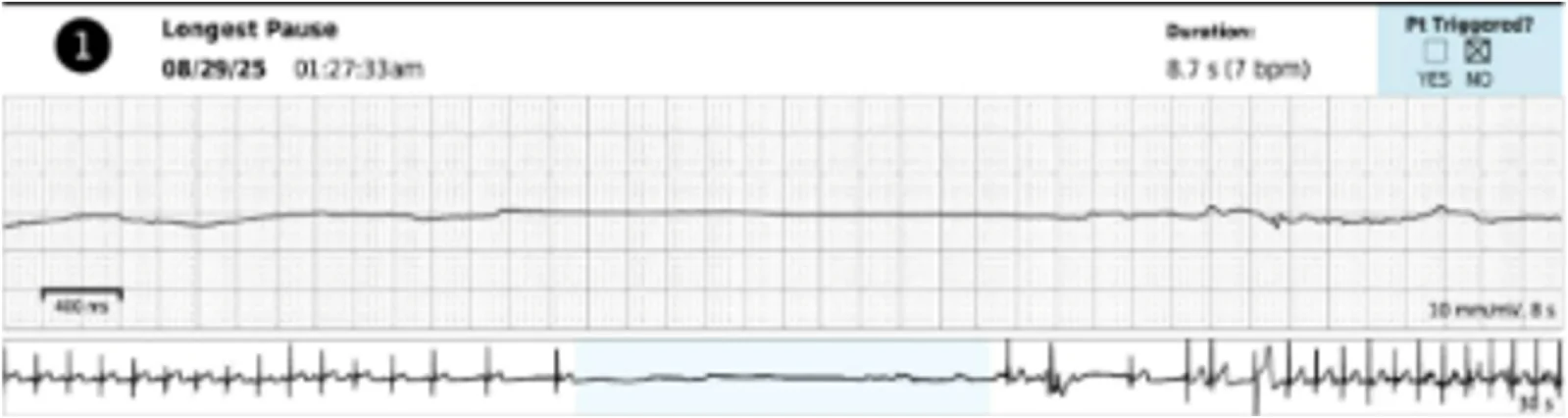
8.7 second sinus pause captured during 14-day Zio patch monitoring during most recent reported syncopal episode.

Given the recurrent nature of her symptoms, she subsequently underwent ambulatory cardiac monitoring with a 14-day Zio patch. The recording captured a single prolonged sinus pause lasting 8.7 seconds during the early morning hours (Fig. 1). Several days after returning the monitor, the patient volunteered that she had fainted during the monitoring period. She described awakening during the night to use the bathroom, experiencing dizziness and abdominal discomfort after urinating, and then losing consciousness. She recalled awakening on the floor moments later. The timing of her account corresponded to the pause captured on the monitor, suggesting that the recorded event occurred during this nocturnal syncopal episode. The Invitae genetic arrhythmia panel was negative for predisposition to arrhythmia. Laboratory testing was also negative for concerns for thyroid disease or electrolyte imbalance.

**Fig. 2:**
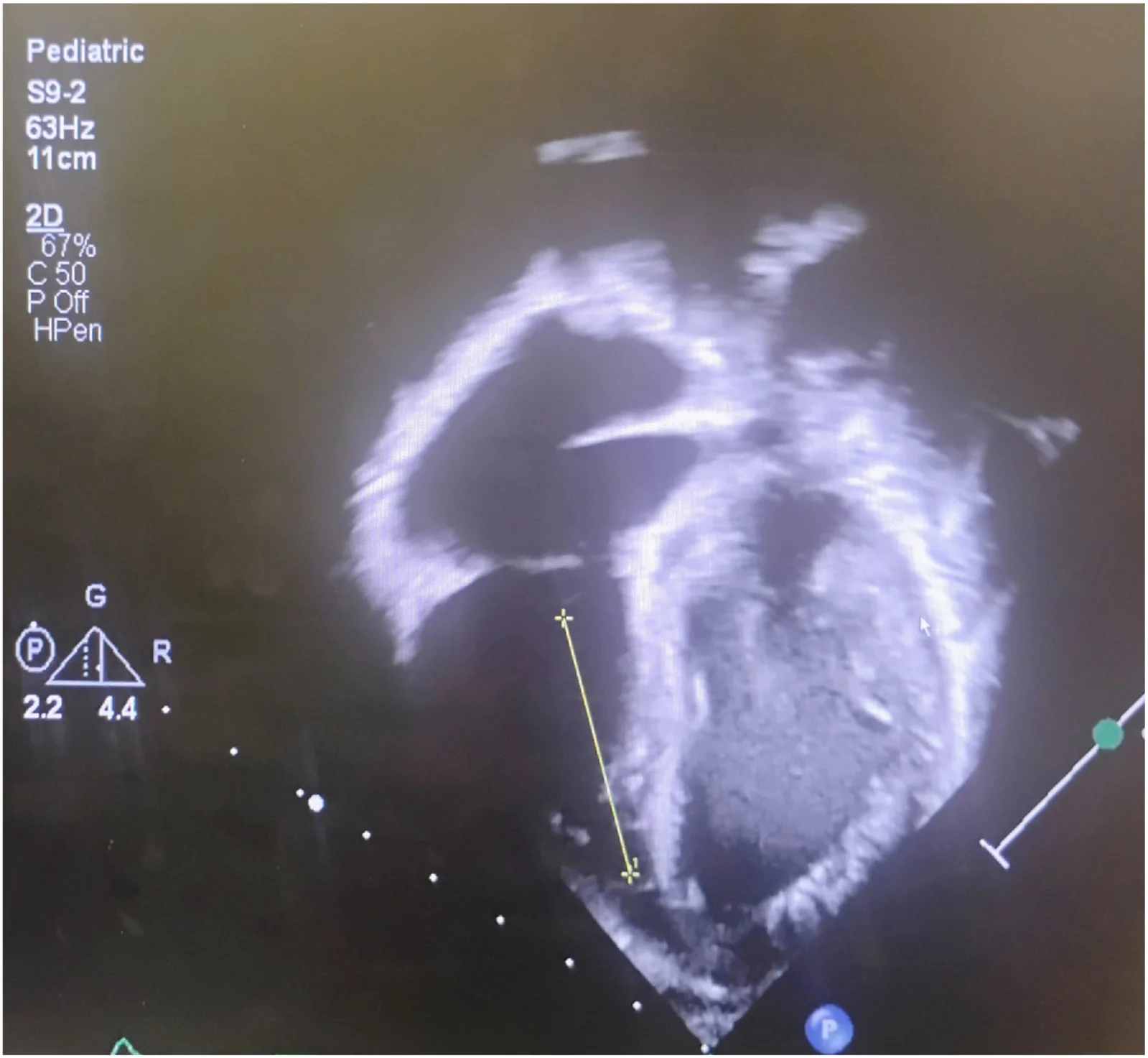
Four chamber apical view via transthoracic echocardiogram prescreening measuring right ventricle dimensions.

Between the events, the patient remained asymptomatic and received a pre-procedure echocardiogram (Fig. 2). She denied palpitations, chest discomfort, or exertional limitation. She was active, kept pace with peers, and reportedly maintained adequate hydration. No additional parental concerns were noted.

## 4 Procedure

Prior to the procedure, the right internal jugular (RIJ), left internal jugular (LIJ), left femoral vein (LFV), and right femoral vein (RFV) were evaluated by ultrasound with Valsalva maneuver, demonstrating vessel diameters of 1 cm for the RIJ, 8 mm for the LIJ, and 7 mm for both femoral veins. Based on vessel caliber, the RIJ was selected given the smaller size of the femoral veins and 27-French sheath needed for access. The Aveir AR was chosen given the shorter length of the device (32 mm versus 38 mm for the Aveir VR, given the patient's small size). After arrival in the procedure suite, the patient underwent airway management and sedation by the anesthesia team. The patient was then prepped and draped in the standard sterile fashion for right internal jugular venous access.

Access to the RIJ was obtained, and a 6-French (Fr) sheath was inserted. A wire was advanced, followed by placement of a 0.035-inch, 180-cm super-stiff Amplatz guidewire through the 6-Fr sheath. Serial dilation was subsequently performed using 8-Fr, to 24-Fr dilators by 2-Fr size. After adequate tract preparation, a 27-Fr (outer diameter) Abbott Aveir sheath, previously flushed, was advanced over the Amplatz wire into the mid–right atrium. The inner sheath was removed, and the outer sheath was connected to heparinized saline.

**Fig. 3:**
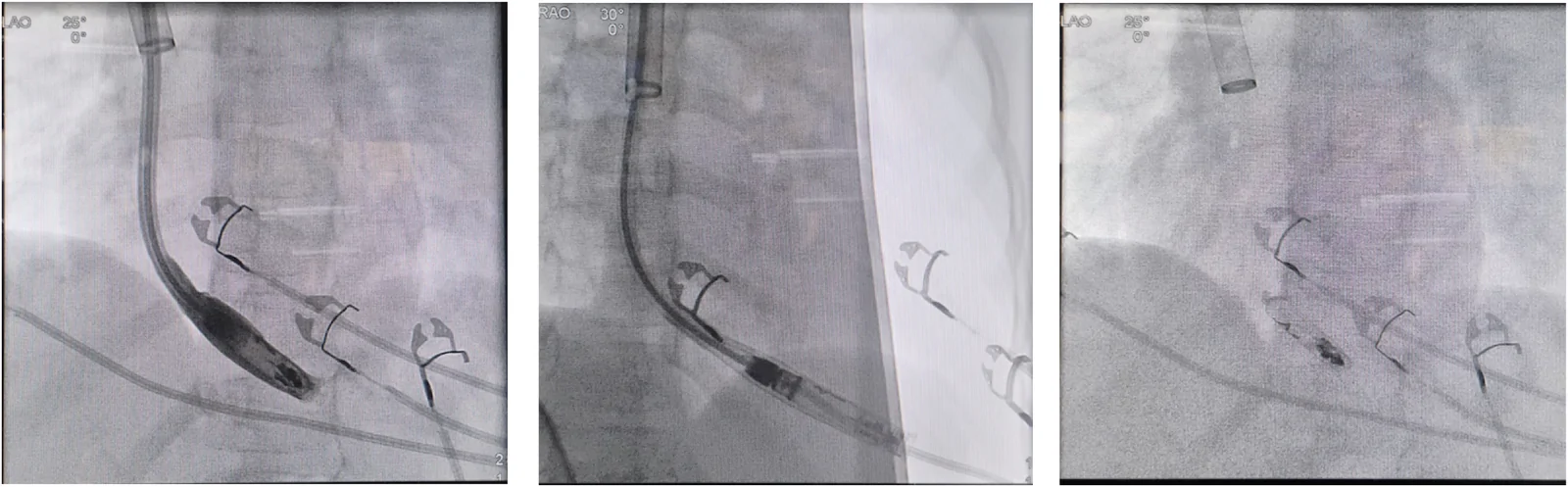
Contrast-enhanced fluoroscopic views of the right ventricle obtained via the right internal jugular approach. (A) Left anterior oblique projection before device fixation. (B) Right anterior oblique projection before device fixation. (C) Left anterior oblique projection after device fixation.

The Aveir device, mounted on a 23-Fr deployment catheter, was then introduced through the 27-Fr outer sheath. The sheath was withdrawn to the junction of the right atrium and superior vena cava (SVC) while maintaining catheter position. The catheter and device were advanced across the tricuspid valve and directed to a mid–right ventricular (RV) septal position. Angiography confirmed an appropriate septal location after contrast injection. The device was successfully deployed on the first attempt, demonstrating satisfactory parameters: pacing threshold 0.5 V at 0.2 ms, R-wave amplitude 4.6 mV, and impedance 400 Ω. A stability test showed persistent positioning with expected movement during catheter deflection. The capture tether was removed and detached. Fig. 3 demonstrates pre- and post-fixation of the device in the right ventricle under fluoroscopy. Transthoracic echocardiography demonstrated stable position with trivial tricuspid regurgitation and no pericardial effusion.

Following device deployment, the 27-Fr sheath was removed, and hemostasis was obtained using a figure-of-eight suture. Additional manual pressure was applied bilaterally, and a pressure dressing was placed over the right internal jugular venous access site.

Post-implant testing demonstrated stable measurements comparable to initial values. The patient was discharged the following day after removal of the neck stitch. One-month follow-up demonstrated a well-healing right internal jugular vein site and device interrogation yielded a ventricular threshold 0.75V at 0.2 ms, impedance 410 Ω, R-wave amplitude 8.3 mV, and the device was programmed at VVI 40 bpm with less than 1% pacing noted and no further syncope. Predicted longevity was 15.9 years.

## 5 Discussion

This case represents the first documented use of the Aveir AR leadless pacemaker implanted in the right ventricle. Leadless pacemakers have continued to evolve in response to limitations of traditional transvenous systems including infection risk, lead fractures, and cosmetic concerns, and their use in pediatric patients has expanded accordingly [7]. However, the unique anatomical constraints of children necessitate individualized and innovative procedural strategies. These decisions align with existing pediatric device guidance, which emphasizes individualized risk–benefit assessment and lifelong pacing needs in children [6].

In this patient, the small length of the retrievable AR device was beneficial given its 32 mm length (compared to 38mm length of the Aveir VR) and reduced size of the right ventricle. The superior approach for Aveir AR implantation was selected for several reasons but primarily due to patient size. Although both the Aveir and Micra systems provide VVI pacing, their battery longevity differs considerably: approximately 12 years for Micra versus a reported 17.6 years for Aveir VR [1]. Extended battery life is particularly advantageous in pediatric populations, as it may reduce the need for device retrieval and replacement during periods of pubertal growth and hormonal variability, when autonomic instability may further exacerbate sinus node dysfunction [8]. Additionally, if device retrieval becomes necessary, the fixation—and therefore retrieval—mechanisms differ between the two systems. The Aveir AR utilize a double-helix fixation with secondary sutures, whereas Micra employs four nitinol tines, leading to distinct extraction strategies [1]. The Aveir system incorporates a docking button compatible with a dedicated retrieval catheter, though snare-based retrieval remains possible if the docking button is inaccessible.

The Aveir system permits electrical mapping prior to fixation, allowing for more precise device positioning [1]. Prior to fixation, careful assessment of cardiac chamber dimensions is essential, as the Aveir and Micra devices differ in length and diameter. Micra measures 25.9 mm in length with a 7-mm diameter, whereas the Aveir VR and AR systems measure 32 mm and 38 mm in length, respectively, with a uniform 6.5-mm diameter [1].

Vascular and intracardiac anatomy played a critical role in procedural planning. The right internal jugular vein (1 cm) was the largest available vascular access site compared with the left internal jugular (8 mm) and bilateral femoral veins (7 mm). Given the larger and accommodating size of the right internal jugular vein, a superior approach was necessary. Finally, Superior access not only accommodated the largest vessel but also allowed for more controlled manipulation of the delivery catheter and facilitated optimal tissue contact for device fixation.

## 6 Conclusion

An Aveir AR leadless pacemaker can be successfully implanted in the right ventricle of a pediatric patient using a superior venous approach, expanding device and access options for children.

## Patient consent page

Written informed consent for publication of this case report and accompanying images was obtained from the patient's legal guardian.

## Ethical consent

This case was approved by the Institutional Review Board of the University of California, Davis, which waived the requirement for formal research consent.

## Declaration of competing interest

Dr. Cortez proctors for Abbott and participates as a consultant for their educational programs. However, no funding was used in the generation of this manuscript.

## References

[1] Saleem-Talib S., Hoevenaars C.P.R., Molitor N., van Driel V.J., van der Heijden J., Breitenstein A. (2025). Leadless pacing: a comprehensive review. Eur Heart J.

[2] Pandya L., Cooper M., Patel N., Leonard D., Fernandes N., Spear D. (2022). Point-of-care ultrasound for central venous assessment in the emergency department: a prospective study comparing the femoral and internal jugular veins. Pediatr Emerg Care.

[3] Neuzil P., Exner D.V., Knops R.E., Cantillon D.J., Defaye P., Banker R. (2025). Worldwide chronic retrieval experience of helix-fixation leadless cardiac pacemakers. J Am Coll Cardiol.

[4] Jacon P., Benkilani M., Bisson A., Marijon E., Da Costa A., Haddad C. (2025). Micra leadless pacemaker revisions: incidence, characteristics, and outcomes from a multicenter French cohort. Europace.

[5] Funasako M., Hála P., Janotka M., Šorf J., Machová L., Petrů J. (2024). Transcatheter non-acute retrieval of the tine-based leadless ventricular pacemaker. Europace.

[6] Shah M.J., Silka M.J., Silva J.N.A., Balaji S., Beach C.M., Benjamin M.N. (2021). 2021 PACES expert consensus statement on the indications and management of cardiovascular implantable electronic devices in pediatric patients. Heart Rhythm.

[7] Crossley G.H., Piccini J.P., Longacre C., Higuera L., Stromberg K., El-Chami M.F. (2023). Leadless versus transvenous single-chamber ventricular pacemakers: 3-Year follow-up of the Micra CED study. J Cardiovasc Electrophysiol.

[8] Coupal K.E., Heeney N.D., Hockin B.C.D., Ronsley R., Armstrong K., Sanatani S. (2019). Pubertal hormonal changes and the autonomic nervous system: potential role in pediatric orthostatic intolerance. Front Neurosci.

